# Telerehabilitation as a Method for Achieving Competencies in Physical and Rehabilitation Medicine Residency Training in a Developing Country: A Protocol for a Pilot Mixed-Methods Study

**DOI:** 10.3389/fresc.2022.921558

**Published:** 2022-05-31

**Authors:** Carl Froilan D. Leochico, Frances Ann B. Carlos, Anna Cecilia S. A. Tiangco, Isabella E. Supnet, Sharon D. Ignacio, Jose Alvin P. Mojica, Reynaldo R. Rey-Matias

**Affiliations:** ^1^Department of Rehabilitation Medicine, College of Medicine, Philippine General Hospital, University of the Philippines Manila, Manila, Philippines; ^2^Department of Physical Medicine and Rehabilitation, St. Luke's Medical Center, Global City, Philippines; ^3^Department of Physical Medicine and Rehabilitation, St. Luke's Medical Center, Quezon City, Philippines

**Keywords:** practice management, education, residency training, telehealth, telemedicine, telerehabilitation, COVID-19, rehabilitation medicine

## Abstract

**Background:**

In the second year of the COVID-19 pandemic, Physical and Rehabilitation Medicine (PRM) residents in a developing country continue to face a lack of in-person clinical exposure and learning opportunities. With the unprecedented shift to virtual care, it remains uncertain whether residents can achieve PRM competencies using telerehabilitation as a method of instruction.

**Objective:**

To determine the PRM residents' ability to achieve competencies through telerehabilitation, as perceived by different stakeholders (residents, chief residents, training officers, and department heads).

**Methods:**

This will be a pilot mixed-methods study, employing concurrent triangulation, in the Department of Rehabilitation Medicine in one large private medical center and one large government hospital in Manila, Philippines. There will be two phases of online data collection upon approval by their respective research ethics board. The first phase will involve an online Likert-scale questionnaire to obtain the residents' self-perceived attainment of competencies and learning of PRM topics and skills specified by the International Society of Physical and Rehabilitation Medicine and the Philippine Board of Rehabilitation Medicine. The results of the survey will then be summarized and presented in a focus group discussion (FGD) with the department heads, training officers, and chief residents of the two institutions in an attempt to explain the residents' perceptions on their competencies achieved through virtual care. Afterwards, the qualitative data obtained from the FGD will then be thematically analyzed, and mixed methods integration will be employed to generate knowledge and recommendations.

**Discussion:**

It is hypothesized that the majority of the residents had little to no experience with telerehabilitation pre-pandemic. Suddenly telerehabilitation was used to augment clinical training during the pandemic. It is uncertain whether telerehabilitation can help residents achieve competencies in the different domains of training, namely: patient safety and quality patient care; medical knowledge and procedural skills; interpersonal and communication skills; practice- and systems-based learning and improvement; reintegration of people with disabilities into the society; medical ethics and public health; quality assurance; policies of care and prevention for disabled people; and professionalism. The study results can provide insights on the aspects of a PRM curriculum that may have to be modified to ensure the training program is sensitive and appropriate to the changing training needs of the residents amid the pandemic and similar crises that may disrupt in-person clinical encounters in the future.

## Introduction

The sudden change in the landscape of Physical and Rehabilitation Medicine (PRM) residency training during the coronavirus disease 2019 (COVID-19) pandemic has caught its traditional curriculum (i.e., heavily reliant on in-person instruction) unprepared for the virtual mode of clinical teaching in various regions worldwide, especially wherein telehealth was not common. In the Philippines, which is a lower middle-income country that had one of the longest COVID-19 lockdowns (1), telerehabilitation was new to the majority of rehabilitation professionals, including physiatrists (2). Telerehabilitation, a subset of telehealth, is defined as “the delivery of rehabilitation services via information and communication technologies,” enabling patient assessments and interventions from a distance (virtual care) (3). There is a growing evidence of telerehabilitation feasibility, effectiveness, safety, and user satisfaction for various disabling conditions, albeit more robust studies are necessary (4). Moreover, telerehabilitation also has an increasing role in providing experiential education to trainees faced with the lack of in-person patient encounters during the pandemic (5, 6).

Indeed, the COVID-19 pandemic has generally highlighted online learning in undergraduate and graduate health professional education; however its carry-over to actual practice remains understudied particularly for programs relying heavily on clinical training (7–9). Studies on students show that nothing can seem to replace seeing a patient in-person to develop clinical skills (7, 9). Nonetheless, given the urgency of the pandemic, some clinical training programs may have developed their guidelines for implementing and supervising virtual care (5, 6, 9), but there is a paucity in the literature regarding the success of these programs in ensuring the students achieve their intended clinical learning outcomes.

In the early part of the pandemic, the Philippine Academy of Rehabilitation Medicine (PARM) released interim guidelines on telerehabilitation to guide physiatrists toward incorporating virtual care in their practice and clinical teaching to ensure safety amid the pandemic (10). To address the lack of cases seen by PRM residents, the different training institutions had come up with various stop-gap measures applicable to their respective hospital policies and capacities. Among the temporary solutions of some institutions was telerehabilitation to help residents meet the required number of clinical hours, caseloads, and academic activities required by their respective hospitals and the Philippine Board of Rehabilitation Medicine (PBRM).

Because of the unprecedented transition to virtual or hybrid (i.e., mixed in-person and virtual) care, it is uncertain whether PRM residents are able to achieve the competencies expected of them based on the original pre-pandemic curriculum, which did not include telerehabilitation. There may be a need to revisit the curriculum of our respective training programs and determine which of its aspects (e.g., learning outcomes, topics, teaching-learning methods) have to be modified to incorporate virtual care and other online activities.

Therefore, the present study aims to answer the following research question: Are PRM residents able to achieve their competencies through telerehabilitation, as perceived by different stakeholders (residents, chief residents, training officers, and department heads) in two training institutions in a developing country that have suddenly incorporated virtual care in their program? The results of the study can guide these institutions, the national specialty board, and other PRM training programs around the world in revising the curriculum to ensure that it is sensitive and appropriate to the changing learning needs of trainees and flexible enough to stand the COVID-19 pandemic and similar crises that may disrupt in-person clinical training in the future.

## Methods

### Research Design

A mixed-methods study design will be employed as part of a larger study aimed at developing a guide in using telerehabilitation as a teaching-learning tool to help PRM residents achieve their competencies. In this concurrent triangulation study, both quantitative and qualitative data will be obtained to provide complementary perspectives on the gaps in learning through telerehabilitation among PRM residents.

### Study Population and Sampling Design

The study population will be a purposive sampling (total enumeration) of the following: (1) all current bona fide PRM resident trainees across all 3-year levels at St. Luke's Medical Center - Quezon City (SLMC-QC) (*n* = 5) and Philippine General Hospital (PGH) (*n* = 24); (2) chief residents (*n* = 2); (3) training officers (*n* = 2); and (4) heads (*n* = 2) of the Department of Rehabilitation Medicine in the aforementioned hospitals. Inclusion criteria will include the following: (1) electronic informed consent; and (2) personal gadget with access to the Internet to be able to respond to an e-survey (i.e., Google Form™) or participate in an online focus group discussion (FGD) (i.e., Zoom™). Exclusion criteria will include the following: (1) on leave from residency training at the time of study; and (2) self-reported to be physically or psychologically unwell during the period of data collection. Withdrawal criteria will include the following: (1) unstable Internet connectivity despite several attempts resulting in inability to either submit responses to the online form or participate in the FGD; and (2) upon request by the FGD participant for any reason at all.

This will be a pilot study, as only two out of the six PRM residency training institutions in the Philippines will be included. The reasons for choosing PGH are as follows: (1) it is the largest government university hospital and longest-running PRM residency training institution in the country with the highest number of residents and graduates; (2) it can elicit unique experiences being a COVID-19 referral center; and (3) it has an established telerehabilitation service program since pre-pandemic. Meanwhile, the reasons for choosing SLMC-QC are as follows: (1) it is the largest and youngest private medical center offering PRM residency training in the country; and (2) its residents engage in telerehabilitation consultations for social service patients. A sample size computation is not deemed necessary for this study.

### Data Collection Plan and Analysis

Letters to obtain permission to recruit participants will be sent to the department head of each of the two involved PRM residency training programs. Once approval is obtained, an e-mail containing the study details and informed consent form will be forwarded through the heads and chief residents. After the potential respondents have signed the consent form, data collection will commence.

There will be two phases of data collection. The first phase will only include the PRM residents as respondents to the online survey (Supplementary Material 1), which consists of the following items:

(1) Demographics: age, sex, highest level of experience with telerehabilitation prior to the pandemic, residency training institution, year level;
(2) Self-perceived attainment of PRM competencies through telerehabilitation; and
(3) Self-perceived learning of PRM topics and skills through telerehabilitation.

The original Likert-scale items for the self-evaluation of competencies and learning contents are based on the review of related literature and documents from the International Society of Physical and Rehabilitation Medicine (ISPRM) and the Philippine Board of Rehabilitation Medicine (PBRM). The questionnaire will undergo pretesting to ensure clarity of questions. The pretest will be conducted on 10 residents randomly recruited from PGH (*n* = 8) and SLMC-QC (*n* = 2). The residents' feedback on the wording, understandability, and applicability of the items will be considered in improving the questionnaire. The final version of the questionnaire will be available on Google Form™ in two separate parts to divide the length of the entire survey, and the links to each form will be distributed to the residents' personal email addresses and also coursed through their private social media groups (e.g., related to department concerns, or residents' group chat), if permitted by the department heads. The residents will be given 2 weeks to answer the 2 parts. The entire survey can be completed for <30 min during their most convenient time. Once a week, the residents will be reminded by the study authors through the help of the chief residents to accomplish the survey, if not yet done. Descriptive statistics (e.g., medians, frequencies, percentages) will be used to summarize the survey results and presented in data tables (Supplementary Material 2).

Aside from the demographic profile, the first part of the survey contains items on the residents' self-perceived attainment of PRM competencies, grouped into the following domains: patient safety and quality patient care; medical knowledge and procedural skills; interpersonal and communication skills; practice- and systems-based learning and improvement; reintegration of people with disabilities into the society; medical ethics and public health; quality assurance; policies of care and prevention for disabled people; and professionalism (11). Each item regardless of domain can be answered by a Likert scale as follows: [4] Strongly Agree; [3] Agree; [2] Disagree; [1] Strongly Disagree; or [0] Not applicable. Each item and domain will be summarized by counting the number of per-item and summed per-domain responses corresponding to each option in the scale. Meanwhile, the second part of the survey contains items on the residents' self-evaluation of learning of PRM topics and skills based on the recommendations of the ISPRM Education Committee (11). Each item is answerable by the following Likert scale: [3] Demonstrated—able to perform skills without supervision; or able to apply theoretical knowledge in PRM practice; [2] Practiced—able to perform skills, but needs supervision; or needs guidance to apply theoretical knowledge in PRM practice; [1] Introduced—able to recognize the principles and processes of PRM skills; or able to discuss PRM theories and concepts; or [0] Not applicable. The frequencies and percentages will also be presented per item, and a subtotal per main topic will present the sum of responses per option.

The second phase of data collection will include the department head, training officer, and chief resident of the 2 institutions (*n* = 6). They will be invited to one FGD through Zoom™. The projected duration of the FGD will be 2–3 h. If the discussion will extend longer than expected, the participants can choose to stay or withdraw at any time and for any reason, but their inputs during the discussion will still be considered in the analysis with their permission. Two study authors will facilitate the FGD. For a smooth conduct of the FGD, a set of predetermined guide questions will be used based on the quantitative results of the survey (e.g., “how can we explain the findings of the survey?,” “why do we think the residents felt that [a certain competency] was most or least achievable through telerehabilitation?,” “which PRM competencies and topics can be adequately taught through telerehabilitation?”), review of the related literature, and anecdotal experiences of the residents and faculty from the study institutions. All the other study co-authors will be present during the FGD to support and balance the different points of view. The teleconference will be recorded solely for data transcription, as indicated in the informed consent form. Three study authors (CDL, FBC, and IES) will review the transcribed data to ensure quality and veracity, and perform thematic analysis. Their subjectivity will be acknowledged from the start of the analysis “to avoid affecting the integrity of the qualitative analysis trajectory” (8). The NVivo software version 12 plus (QSR International Pty Ltd., VIC, Australia) will be used to code the data and categorize relevant text fragments into themes (Supplementary Material 3).

**Figure 1:**
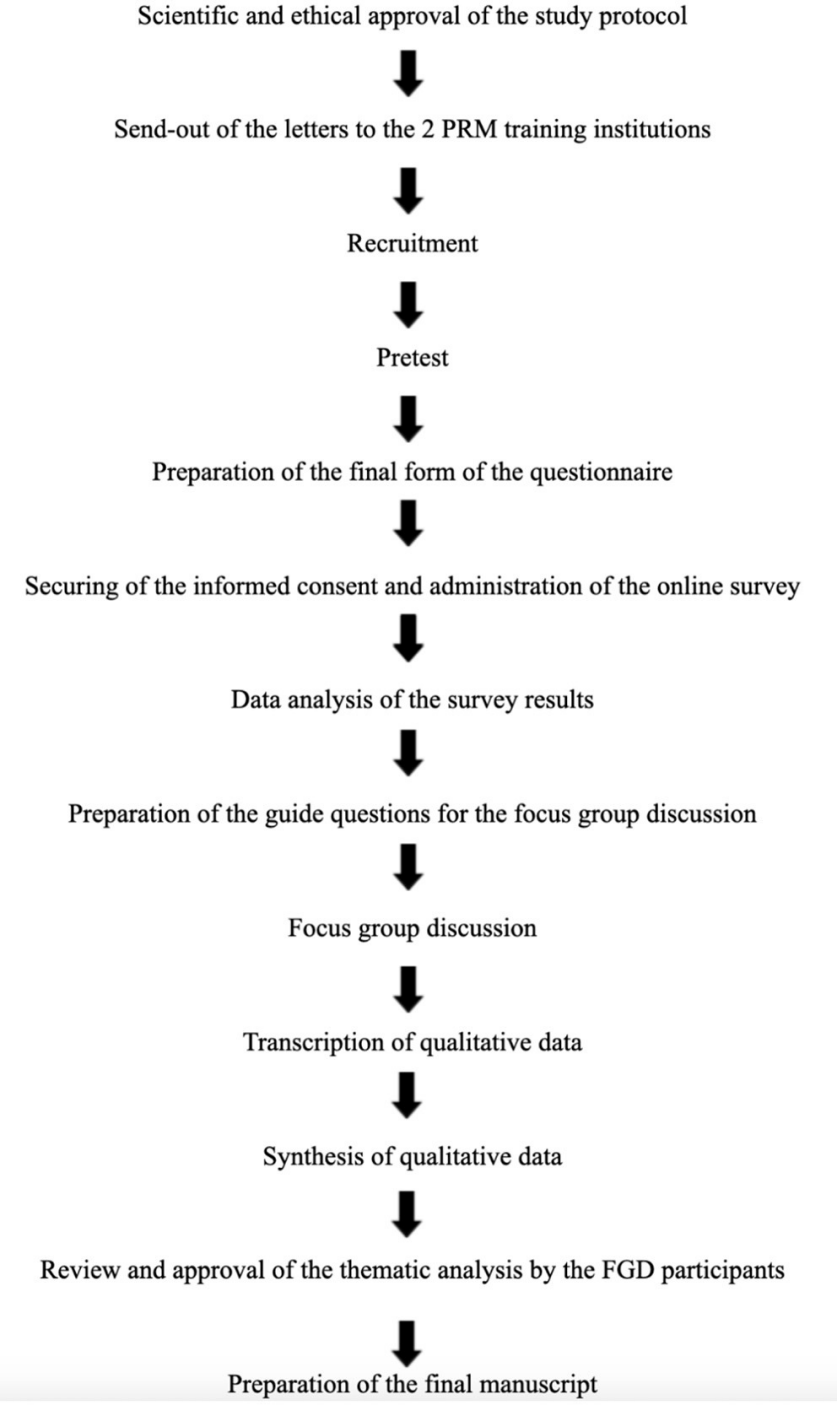
Study procedure.

Afterwards, findings from the quantitative and qualitative phases of data collection will be analyzed by the study authors through mapping of data onto each other and carefully reflecting upon them. Employing mixed methods integration, an iterative joint display (i.e., meta matrix) analysis process will be used to generate meta inferences (8). Finally, the study authors will compare and contrast findings from the quantitative and qualitative data analyses to develop a meaningful narrative and set of recommendations on possible curricular revisions to incorporate telerehabilitation in PRM residency training. The final thematic analysis of the FGD and recommendations will be forwarded to the FGD participants for review and approval. The flow of data collection and analysis is summarized in Figure 1.

## Ethical Considerations

The following guidelines will be used in developing the completed paper: (1) Strengthening the Reporting of Observational Studies in Epidemiology (STROBE) statement for reporting of quantitative data; and (2) Standards for Reporting Qualitative Research for reporting of qualitative data. The study protocol will undergo review by the University of the Philippines Manila—Research Ethics Board (UPM-REB) at PGH and the scientific and ethical committees of the Research and Biotechnology Group of SLMC-QC.

All quantitative data to be collected from the survey will remain anonymous through unique identifiers (i.e., participant code number), while data from the FGD will be transcribed with pseudonyms to be given discretely by each participant, whose real name, position and institution will remain undisclosed throughout the data analysis and manuscript writing. There is no foreseeable risk associated with the study participation, while its benefits are manifold as follows: (1) contribution to the development of improvement strategies for residency training; (2) opening the platforms for discussion of unexpressed feelings toward the current residency training program; and (3) provision of concrete recommendations to potentially incorporate a formal telerehabilitation curriculum into PRM residency in the Philippines, if deemed necessary based on the results of the study. There will be no vulnerable subjects and minors involved in the study.

No form of remuneration will be given to the survey and FGD participants. The envisioned long-term benefit that the study can contribute to the community will be the quality improvement of telerehabilitation services and PRM residency training in the country. The study aims and methods are not foreseen to cause any negative effect on the community.

The results of the study will be shared with the 2 involved training institutions and the Philippine Board of Rehabilitation Medicine. The completed study will be presented in local and international scientific conferences for sharing or exchange of experiences, and submitted to an international reputable journal in PRM.

## Discussion

This pilot study will provide baseline information on the PRM residents' ability to achieve core competencies through telerehabilitation. It is hypothesized that the majority of the residents are familiar with telerehabilitation and its technical know-how, but may have limited skills and experience in using it for virtual care. Suddenly telerehabilitation was used for patient encounters during the pandemic. Hence, it is uncertain whether telerehabilitation as a teaching-learning tool can help PRM residents achieve competencies in the different domains of training identified by the ISPRM, which is the internationally recognized society of the specialty.

Possible variations in the responses of the residents may be observed in the survey results based on their training institution, year level, and telerehabilitation experience pre-pandemic. In the future, the results of this pilot study can be used to come up with a larger study that involves all the other PRM training institutions in the Philippines. Nonetheless, it may be surmised that the residents may be able to improve their interpersonal and communication skills, medical knowledge, and even procedural skills through virtual patient encounters and didactics, as these are among the competencies expected in a telemedicine curriculum (12). In addition, through virtual care the residents may also be able to attain competencies in the other domains, such as patient safety and quality patient care, practice- and systems-based learning and improvement, reintegration of people with disabilities into the society, medical ethics and public health, quality assurance, policies of care and prevention for disabled people, and professionalism.

The residents' knowledge on the core topics recommended by the ISPRM, which is followed by the local PBRM, can be enhanced through telerehabilitation, although the skills-based competencies may have to supplemented with hands-on training and repeated and supervised in-person patient encounters. In a study by Chiu et al. the majority of the residents reported to have lesser clinical experience and lesser interaction with and supervision from attending physicians during telemedicine compared to in-person consultations (13). Meanwhile, practicing physiatrists in the Philippines expressed concerns about the lack of thorough patient examination through telerehabilitation (2).

The results of this study can catalyze the next steps toward curricular improvements in PRM residency training. Specifically, knowing which aspects of the pre-pandemic curriculum can be taught through telerehabilitation can guide PRM educators and administrators in optimizing telerehabilitation as a teaching-learning method for residency training, augmenting traditional in-person mode of instruction. Curricular improvements can also consider the inclusion of PRM competencies specific to telemedicine, such as standard virtual communication, webside manners, remote physical examination techniques, utilization of various telemedicine technology platforms, and skills in documenting virtual encounters (12). These competencies may equip the modern generations of PRM graduates for a more competent and professional conduct of telerehabilitation, which can be leveraged especially during national or international crises disrupting in-person delivery of rehabilitation services and clinical training.

## Ethics Statement

The authors confirm that the manuscript has neither been previously published nor currently being considered for publication elsewhere. The manuscript reflects the authors' own research work. All sources used are properly cited. All authors have contributed substantially to the paper and will take public responsibility for its content.

## Author Contributions

CL conceptualized the research idea and wrote the protocol draft and revisions. FC, AT, and IS participated in the literature review and supplied details in different parts of the protocol. All authors contributed to the development of the research idea and approved the submitted version.

## Conflict of Interest

The authors declare that the research was conducted in the absence of any commercial or financial relationships that could be construed as a potential conflict of interest.

## Publisher's Note

All claims expressed in this article are solely those of the authors and do not necessarily represent those of their affiliated organizations, or those of the publisher, the editors and the reviewers. Any product that may be evaluated in this article, or claim that may be made by its manufacturer, is not guaranteed or endorsed by the publisher.
