# Supplementary material for: Telerehabilitation as a Method for Achieving Competencies in Physical and Rehabilitation Medicine Residency Training in a Developing Country: A Protocol for a Pilot Mixed-Methods Study
Source: Front Rehabil Sci. 2022 May 31;3:921558. doi: 10.3389/fresc.2022.921558 (PMC9397763; doi:10.3389/fresc.2022.921558)
Supplement: Supplementary file 1 [file Table_1.docx]

**SUPPLEMENTARY MATERIALS**

**Supplementary Material 1: Survey Form**

Part 1. Demographic Profile

| **Age (years)** |  |
| --- | --- |
| **Sex** | - Male - Female |
| **Prior to the pandemic, what was your highest level of experience with telerehabilitation?** | - Was able to perform telerehabilitation with actual patients - Was able to witness, but not perform, telerehabilitation with actual patients - Was able to adequately learn about telerehabilitation principles and techniques - Was able to hear about telerehabilitation, but not able to adequately learn about telerehabilitation principles and techniques - Never heard of telerehabilitation |
| **Residency training institution** | - Philippine General Hospital - St. Luke’s Medical Center – Quezon City |
| **Current year level in residency training** | - 1^st^ - 2^nd^ - 3^rd^ |

Part 2. Self-evaluation of PRM Competency Attainment through Telerehabilitation

Below are the competencies that a resident is expected to achieve by the end of the Physical and Rehabilitation Medicine (PRM) training program. Based on your honest self-reflection and personal experiences with telerehabilitation, please evaluate each item according to the following rating:

[4] Strongly Agree;

[3] Agree;

[2] Disagree;

[1] Strongly Disagree;

[0] Not applicable.

(All items below are based on the Core Curriculum and Competency recommended by the International Society of Physical and Rehabilitation Medicine and the Philippine Board of Rehabilitation Medicine, 2017: https://www.isprm.org/wp-content/uploads/2019/06/DRAFT-PRM-Core-curriculum-Competency-Final-28.06_.pdf)

| *Domain* | *Survey item as it relates to telerehabilitation experience* |
| --- | --- |
| **Patient Safety and Quality Patient Care** | - Provide patient care that is compassionate, appropriate, and effective for the treatment of health problems and promotion of health. - Demonstrate competence in the evaluation and management of patients with physical and/or cognitive impairments, disabilities, and functional limitations across different age groups. - Understand and provide appropriate prescription/ consultation for evaluation and management by other rehabilitation professionals (e.g. Physical therapy, Occupational therapy, Speech/ language pathologist, Therapeutic/ recreational specialist, Psychologists and vocational counselors, Rehabilitation nurse), while overseeing and monitoring the rehabilitation program. - Work in interprofessional teams. - Coordinate effectively and efficiently with an interdisciplinary team of allied rehabilitation professionals for the maximum benefit of the patient through:   - a.) an understanding of each allied health professional's role.   - b.) the ability to determine precise rehabilitation goals and prescribe adequately detailed rehabilitation prescriptions, towards functional recovery/outcome, considering prognosis, physical, environmental, and social factors.   - c.) the development of management and leadership skills. - Be able to assess the needs of a patient hospitalized in an acute care facility and suggest an adequate treatment/ recommendation. - Organize admission to a rehabilitation facility. - Organize the discharge from a rehabilitation facility. - Establish and coordinate measures for disability compensation. - Have experience in the continuing care of patients with long-term disabilities through appropriate follow-up care. |
| **Medical Knowledge and Procedural Skills** | - Demonstrate understanding of the pathophysiologic aspects, risk factors and functional prognosis of disorders in PRM, and describe the deficiencies, activity limitations, and participation restrictions as consequences of such disorders. - Utilize appropriate diagnostics and assessments, both clinical and technical means, to explore functions, with eventual development of a rehabilitation management plan using pharmacologic and non-pharmacologic, physical, cognitive, and behavioral treatments, as well as means for disease prevention. - Independently perform comprehensive and specific physiatric examinations. - Perform diagnostic and treatment procedures common to the practice of PRM, such as electrodiagnostic medicine, MSK ultrasonography, and peripheral and axial injections, with either synchronous or asynchronous remote guidance by a consultant. - Prescribe different kinds of exercise. |
| **Interpersonal and Communication Skills** | - Demonstrate interpersonal and communication skills that result in effective exchange of information and collaboration with patients, their families, and other health professionals. - Exhibit effective and appropriate communication with patients, families, and the public across different socioeconomic and cultural backgrounds. - Work effectively as a member or leader of a healthcare team or other professional group, and act as a consultative role to other physicians or health professionals. - Maintain comprehensive, timely, and understandable medical records. |
| **Practice- and Systems-Based Learning and Improvement** | - Observe and gain fundamental understanding of the types of patients served, referral patterns and services available in the continuum of rehabilitation care provided in various settings. These may include critical, acute, and sub-acute care units, skilled nursing facilities, sheltered workshops, and other vocational facilities, schools for persons with multiple disabilities (including deafness and blindness), independent living facilities for individuals with physical impairments, day hospitals, home health care services, primary care setting, as well as community-based rehabilitation. Introduction to these options for care may be made by on-site visits to some of these facilities as well as didactic lectures. Residents should be encouraged to interact with health care consumer groups and organizations in supervised working environments. - Identify the inclusion criteria for a physical/ cognitive rehabilitation program for a patient and criteria for discharge. - Identify the main relevant patient groups for a disabled person. - Read pertinent articles from recent evidence-based medical literature on the assessment and treatment of conditions seen in Rehabilitation Medicine and apply this knowledge to the current care of a patient. - Use information technology to appraise and assimilate evidence from scientific studies to enhance patient care outcomes. - Engage in and write a research under supervision and disseminate research findings. - Present a report/ lecture to the consultants, residents, and other members of the rehabilitation team on an aspect of interest in the assessment and treatment of conditions seen in Rehabilitation Medicine. - Discuss with the consultants, residents, and other members of the rehabilitation team the limitations in the current knowledge base of a particular condition and possible directions for future clinical research to improve knowledge in this area. - Demonstrate skill in supervising residents, medical students, and members of the rehabilitation team in information gathering, decision-making and patient management. |
| **Reintegration of People with Disabilities into Society** | - Identify resources of education and training for a disabled person and/or participate in the orientation. - Identify resources of professional rehabilitation and/or participate in the orientation for reintegration. - Advocate for quality patient care and optimal patient care systems. - Identify the health, social and financial barriers, and possible resources. - Identify and establish the means allowing a disabled person to remain at home. |
| **Medical Ethics and Public Health** | - Identify individual and collective issues of public health and ethics related to disabled people. - Identify clinical situations (during rehabilitation) of unreasonable obstinacy related to care. - Conduct multi-professional discussions aiming at care limitations with the patient and relatives/ caregivers. - Incorporate considerations of cost awareness and risk-benefit analysis in patient and/or population-based care as appropriate. |
| **Quality Assurance** | - Participate in identifying system errors and implementing potential systems solutions. - Receive formal instruction regarding the principles, objectives and process of performance improvement and program evaluation, risk management, and cost-effectiveness in Medicine |
| **Policies of Care and Prevention for Disabled People** | - Log PRM clinical activities and practical procedures. - Participate in public information about prevention and care for the main disabling diseases and the social integration of disabled people. |
| **Professionalism** | - Demonstrate a commitment to carry out professional responsibilities and an adherence to ethical principles. - Demonstrate compassion, integrity, and respect for others - Respect for patient privacy and autonomy. - Demonstrate responsiveness to patient needs that supersedes self-interest. - Demonstrate accountability to patients, society, and the profession. - Be sensitive and responsive to a diverse patient population, including, but not limited to diversity in gender, age, culture, race, religion, disabilities, and sexual orientation. |

Part 3. Self-evaluation of Learning of PRM Topics and Skills through Telerehabilitation

Below are the specific topics and skills that a resident is expected to learn by the end of the Physical and Rehabilitation Medicine (PRM) training program. Based on your honest self-reflection and personal experiences with telerehabilitation, how far have you learned the following items during your telerehabilitation sessions:

[3] Demonstrated – able to perform skills without supervision; or able to apply theoretical knowledge in PRM practice;

[2] Practiced – able to perform skills, but needs supervision; or needs guidance to apply theoretical knowledge in PRM practice;

[1] Introduced – able to recognize the principles and processes of PRM skills; or able to discuss PRM theories and concepts; or

[0] Not applicable.

(All items below are based on the Core Curriculum and Competency recommended by the International Society of Physical and Rehabilitation Medicine and the Philippine Board of Rehabilitation Medicine, 2017: https://www.isprm.org/wp-content/uploads/2019/06/DRAFT-PRM-Core-curriculum-Competency-Final-28.06_.pdf)

| **TOPIC** | ***Learning content as it relates to telerehabilitation experience*** | **D** | **P** | **I** | **NA** |
| --- | --- | --- | --- | --- | --- |
| **FUNDAMENTALS** | | | | | |
| **Rehabilitation Goals** | Broad definition of rehabilitation goals |  |  |  |  |
|  | Definition of short, medium and long term goals |  |  |  |  |
|  | History & Physical examination in PRM |  |  |  |  |
| **Rehabilitation theoretical background** | Sensory and Motor control systems |  |  |  |  |
|  | Posture and Balance control systems |  |  |  |  |
|  | Motor learning principles |  |  |  |  |
|  | Neuroplasticity |  |  |  |  |
|  | Mirror neuron paradigm |  |  |  |  |
|  | Principles of cardiovascular fitness |  |  |  |  |
|  | Principles of Kinesiology |  |  |  |  |
|  | Mechanisms of Respiration |  |  |  |  |
|  | Mechanisms of Excretion |  |  |  |  |
|  | Mechanisms of Swallowing |  |  |  |  |
|  | Physiology of aging |  |  |  |  |
| **WHO-ICF** | Definition of functioning and health |  |  |  |  |
|  | Classification in detail: Body Structure and Functions, Activity and Participation, Environmental factors, Capacity and Performance concepts |  |  |  |  |
|  | Check-list and Core-sets |  |  |  |  |
|  | ICF-Cy |  |  |  |  |
|  | ICF application to epidemiological research |  |  |  |  |
| **Functional assessment and outcome measurement** | Psychometric properties of clinical measures (accuracy, reliability, validity, feasibility, ceiling and floor effect, transcultural validation) |  |  |  |  |
|  | Classification of clinical outcome measures |  |  |  |  |
|  | (Category, Ordinal, Interval scales) |  |  |  |  |
|  | Pain measurements (VAS, NRS, Mc Gill Pain questionnaire,…) |  |  |  |  |
|  | Range of motion (goniometric measures) assessment |  |  |  |  |
|  | Muscle strength (Medical Research Council scale,…..) assessment |  |  |  |  |
|  | Motor impairment measures (examples, Motricity index, FuglMeyer,….) |  |  |  |  |
|  | Esteroception and proprioception assessment |  |  |  |  |
|  | Cranial nerve assessment |  |  |  |  |
|  | Bedside swallow assessment |  |  |  |  |
|  | ADL measures (examples , Barthel Index, Functional Assessment Measure) |  |  |  |  |
|  | Trunk control assessment (examples, Trunk control test, ….) |  |  |  |  |
|  | Gait measures (examples, Functional Ambulation Category, Hauser index, 10 m walking test) |  |  |  |  |
|  | Balance measures (examples, Tinetti scale, Berg Balance scale, Timed Up and Go) |  |  |  |  |
|  | Endurance measures (Borg scale, 6 m walking test,…) |  |  |  |  |
|  | Dexterity measures (examples: ARA test, Wolf MFT, Nine hole peg test, Motor Activity Log,.) |  |  |  |  |
|  | Cognition assessment: general (examples, Mini Mental state exam, Frontal Assessment battery, ) |  |  |  |  |
|  | Cognition assessment: focal (examples, Memory tests, Attention tests, Visuospatial ability tests, Frenchay Aphasia Screening test …) |  |  |  |  |
|  | Spasticity (examples Modified Ashworth scale, Tardieu scale, Goal Attainment score…..) |  |  |  |  |
|  | Speech |  |  |  |  |
|  | Consciousness Level |  |  |  |  |
|  | Sensory Disturbance |  |  |  |  |
|  | Quality of life (SF-36, Euroqol, …..) |  |  |  |  |
|  | Community reintegration inventories (i.e., Community Integration Questionnaire, Functional Status Questionnaire..) |  |  |  |  |
|  | Disease-specific scales (specific disability assessment for people with Neurological disorders, Musculoskeletal disorders, Cancer ,..) |  |  |  |  |
| **DIAGNOSTICS (knowledge includes: indications to the investigation, interpretation of major findings)** | Ultrasound assessment of joints and muscles |  |  |  |  |
|  | Ultrasound assessment of artero-venous vessels |  |  |  |  |
|  | Skeletal X-ray |  |  |  |  |
|  | Chest and abdominal X-ray |  |  |  |  |
|  | Brain CT |  |  |  |  |
|  | Brain MRI |  |  |  |  |
|  | Spine CT |  |  |  |  |
|  | Spine MRI |  |  |  |  |
|  | Chest and abdominal CT |  |  |  |  |
|  | Scintigram |  |  |  |  |
|  | Bone Mineral Density |  |  |  |  |
|  | EMG-ENG |  |  |  |  |
|  | EEG |  |  |  |  |
|  | Evoked potentials (MEPs, SSEPs, BAEPs,..) |  |  |  |  |
|  | Videofluoroscopy, Fiberoptic endoscopic evaluation of swallowing |  |  |  |  |
|  | Gait analysis (sEMG, kinetics) |  |  |  |  |
|  | Posturography |  |  |  |  |
|  | Motion analysis |  |  |  |  |
| **INTERVENTIONS (indications, efficacy, side effects)** | Education, psychological support to increase resilience/coping with disabilities |  |  |  |  |
|  | Drug treatment – analgesic, opioids, anticonvulsant |  |  |  |  |
|  | Drug treatments- anti-inflammation treatment |  |  |  |  |
|  | Drug treatment – oral and local spasticity treatment (botulinum toxin, phenol nerve blocks) |  |  |  |  |
|  | Drug treatment- antidepressant agents |  |  |  |  |
|  | Drug treatment- excretory disturbance |  |  |  |  |
|  | Therapeutic exercise, aquatherapy |  |  |  |  |
|  | Physical modalities, massage, manipulation, traction |  |  |  |  |
|  | Electrical stimulation (Transcutaneous, Functional /Neuromuscular) |  |  |  |  |
|  | Biofeedback |  |  |  |  |
|  | Neurodevelopmental approaches (Bobath, Kabat, Vojta, Rood,……) |  |  |  |  |
|  | Task-oriented approaches |  |  |  |  |
|  | Neurocognitive approach according to Perfetti |  |  |  |  |
|  | Action observation techniques |  |  |  |  |
|  | Mirror therapy |  |  |  |  |
|  | Non invasive cortical stimulation techniques |  |  |  |  |
|  | Orthotics and prosthetics |  |  |  |  |
|  | Locomotion aids/adaptive equipments (canes, rollators, power/manual wheelchairs, bath equipments) |  |  |  |  |
|  | Assistive technology , Augmentative communication device |  |  |  |  |
|  | Ergonomic Considerations in House, Workplace |  |  |  |  |
|  | Vocational Rehabilitation |  |  |  |  |
|  | Sport therapy |  |  |  |  |
|  | Music therapy, art therapy, pet therapy and other complementary/alternative medicine |  |  |  |  |
|  | Balneology, SPA therapy |  |  |  |  |
|  | Manual medicine |  |  |  |  |
|  | Acupuncture |  |  |  |  |
| **RESEARCH IN REHABILITATION** | Principles of epidemiology, quantitative and qualitative research |  |  |  |  |
|  | Research study design (experimental and observational studies, single-case studies, metanalysis and reviews) |  |  |  |  |
|  | Fundamentals of descriptive statistics (mean, SD, variance, confidence intervals, median, range, interquartile range; normal distribution) |  |  |  |  |
|  | Fundamentals of inferential statistics (independent and dependent variables, comparison statistical analysis for parametric and non parametric measures) |  |  |  |  |
|  | Sample size computation |  |  |  |  |
|  | Reporting results in graphics and tables, narrative assessment of outcome |  |  |  |  |
|  | Quality Improvement |  |  |  |  |
| **REHABILITATION APPROACHES TO DISEASE-SPECIFIC DISABILITIES**  **(Pathogenesis, clinical assessment, rehabilitation techniques, prognostic factors of recovery)** | | | | | |
| **Nervous system disorders** | Stroke |  |  |  |  |
|  | Acquired brain injury in adult age |  |  |  |  |
|  | Acquired brain injury in developmental age |  |  |  |  |
|  | Spinal cord injury (traumatic and not traumatic) in adults |  |  |  |  |
|  | Spinal cord injury (traumatic and not traumatic) in children |  |  |  |  |
|  | Autoimmune and inflammatory neurological conditions (e.g. Multiple Sclerosis) |  |  |  |  |
|  | Movement disorders (as a composite clinical condition) |  |  |  |  |
|  | Parkinson’s disease |  |  |  |  |
|  | Huntington disease |  |  |  |  |
|  | Dystonias |  |  |  |  |
|  | Peripheral Nervous System disorders and injuries (as a composite clinical condition) |  |  |  |  |
|  | Neuropathies |  |  |  |  |
|  | Myopathies/Dystrophies |  |  |  |  |
|  | Neuromuscular diseases in adults (including post-polio syndrome) |  |  |  |  |
|  | Neuromuscular diseases in developmental age (SMA, botulism) |  |  |  |  |
|  | Focal disorders of cognition and behaviour |  |  |  |  |
|  | Language and Speech disorders |  |  |  |  |
|  | Swallowing disorders |  |  |  |  |
|  | Cerebral palsy during the developmental age (including normal growth & development) |  |  |  |  |
|  | Cerebral palsy in the grown subjects |  |  |  |  |
|  | Congenital disorders of Nervous System (e.g. spina bifida, Arnold-Chiari malformation and so on) |  |  |  |  |
|  | Specific learning disorders in children (e.g. dyslexia, ADHD, mental retardation, dyspraxia….) |  |  |  |  |
| **Orthopaedic and Musculoskeletal Disorders** | Osteoarthritis, crystal arthritis and degenerative musculoskeletal conditions |  |  |  |  |
|  | Hand Injuries |  |  |  |  |
|  | Musculoskeletal injuries |  |  |  |  |
|  | Limb loss (including congenital causes) and Amputations |  |  |  |  |
|  | Osteoporosis |  |  |  |  |
|  | Widespread pain syndromes (fibromyalgia, chronic fatigue syndrome, etc) and work-related musculoskeletal disorders/Industrial rehab |  |  |  |  |
|  | Spinal Disorders (including back pain, scoliosis) |  |  |  |  |
|  | Inflammatory and autoimmune disorders, joint & connective tissue disorders (e.g. Rheumatoid Arthritis and SLE, etc.) |  |  |  |  |
|  | Rehabilitation after joint prosthesis |  |  |  |  |
|  | Temporomandibular joint disorders |  |  |  |  |
|  | Sports Medicine |  |  |  |  |
| **Other Specific Disabling Conditions** | Pain management: Complex regional pain syndromes / Central pain / Neuropathic pain disorders/ Craniofacial pain/ Pelvic pain/ |  |  |  |  |
|  | Respiratory Diseases, ventilation failure (Pulmonary Rehabilitation) |  |  |  |  |
|  | Heart diseases (Cardiac Rehabilitation) |  |  |  |  |
|  | Peripheral Artery Diseases |  |  |  |  |
|  | Cancer : Oncological rehabilitation / |  |  |  |  |
|  | The frail patient (including the immobile patient); management of |  |  |  |  |
|  | Deconditioning, DVT prophylaxis/Treatment in Rehab patients |  |  |  |  |
|  | Postural Instability and Recurrent Falls |  |  |  |  |
|  | Wound Care (promotion of tissue viability, prevention and treatment of Pressure Sores) |  |  |  |  |
|  | Bladder and Bowel Disorders (perineal rehabilitation) |  |  |  |  |
|  | Sexual disorders (rehabilitation after spine lesion) |  |  |  |  |
|  | Disability after organ transplantation |  |  |  |  |
|  | Vascular Insufficiency/Dse, Lymphedema |  |  |  |  |
|  | Disability following major burns |  |  |  |  |
|  | Miscellanea (disability linked to severe obesity, metabolic disorders, psychiatric disorders, hearing & visual disorders, …) |  |  |  |  |
| **INTEGRATIVE AND CLINICAL REHABILITATION SCIENCES** | | | | | |
| **Integrative and clinical rehabilitation sciences** | Comprehensive PRM Intervention definition |  |  |  |  |
|  | Administration and management |  |  |  |  |
|  | Research on best care including guidelines, organization, coordination, and education |  |  |  |  |
|  | Standards and guidelines for the provision of best care (including Evidence Based Medicine) in PRM |  |  |  |  |
|  | PRM quality management |  |  |  |  |
|  | Scientific education and training of professionals in PRM |  |  |  |  |
|  | Development and evaluation of the PRM team and multidisciplinary care |  |  |  |  |
|  | Community-based rehabilitation issues |  |  |  |  |
|  | Networks and pathways in PRM |  |  |  |  |
| **Life after residency (for senior trainees)** | Different practice settings /marketing your practice |  |  |  |  |
|  | Medical liability |  |  |  |  |
|  | Interview process |  |  |  |  |
|  | Billing & Collections |  |  |  |  |
|  | Employment contracts |  |  |  |  |

**Supplementary Material 2: Dummy Tables for the Survey Results**

Table 1. Demographic profile of the PRM residents (N = 30).

| ***Characteristics*** | ***n (%) or X (SD)*** |
| --- | --- |
| **Age (years)** | X (SD) |
| **Sex**   - Female - Male | n (%)  n (%) |
| **Prior to the pandemic, what was your highest level of experience with telerehabilitation?**   - Was able to perform telerehabilitation with actual patients - Was able to witness, but not perform, telerehabilitation with actual patients - Was able to adequately learn about telerehabilitation principles and techniques - Was able to hear about telerehabilitation, but not able to adequately learn about telerehabilitation principles and techniques - Never heard of telerehabilitation | n (%)  n (%)  n (%)  n (%) |
| **Residency training institution**   - Philippine General Hospital - St. Luke’s Medical Center – Quezon City | n (%)  n (%) |
| **Current year level in residency training**   - 1^st^ - 2^nd^ - 3^rd^ | n (%)  n (%)  n (%) |

Table 2. Residents’ self-evaluation of PRM competency attainment through telerehabilitation (N =30).

| ***PRM Competencies per Domain**** | ***4*** | ***3*** | ***2*** | ***1*** | ***0*** |
| --- | --- | --- | --- | --- | --- |
| **Patient Safety and Quality Patient Care**   - Provide patient care that is compassionate, appropriate, and effective for the treatment of health problems and promotion of health. - Demonstrate competence in the evaluation and management of patients with physical and/or cognitive impairments, disabilities, and functional limitations across different age groups. - Understand and provide appropriate prescription/ consultation for evaluation and management by other rehabilitation professionals (e.g. Physical therapy, Occupational therapy, Speech/ language pathologist, Therapeutic/ recreational specialist, Psychologists and vocational counselors, Rehabilitation nurse), while overseeing and monitoring the rehabilitation program. - Work in interprofessional teams. - Coordinate effectively and efficiently with an interdisciplinary team of allied rehabilitation professionals for the maximum benefit of the patient through:   - a.) an understanding of each allied health professional's role.   - b.) the ability to determine precise rehabilitation goals and prescribe adequately detailed rehabilitation prescriptions, towards functional recovery/outcome, considering prognosis, physical, environmental, and social factors.   - c.) the development of management and leadership skills. - Be able to assess the needs of a patient hospitalized in an acute care facility and suggest an adequate treatment/ recommendation. - Organize admission to a rehabilitation facility. - Organize the discharge from a rehabilitation facility. - Establish and coordinate measures for disability compensation. - Have experience in the continuing care of patients with long-term disabilities through appropriate follow-up care.   ***Total for the Domain*** | n (%)  n (%)  n (%)  …  $\Sigma$ (%) | n (%)  n (%)  n (%)  …  $\Sigma$ (%) | n (%)  n (%)  n (%)  …  $\Sigma$ (%) | n (%)  n (%)  n (%)  …  $\Sigma$ (%) | n (%)  n (%)  n (%)  …  $\Sigma$ (%) |
| **Medical Knowledge and Procedural Skills**   - Demonstrate understanding of the pathophysiologic aspects, risk factors and functional prognosis of disorders in PRM, and describe the deficiencies, activity limitations, and participation restrictions as consequences of such disorders. - Utilize appropriate diagnostics and assessments, both clinical and technical means, to explore functions, with eventual development of a rehabilitation management plan using pharmacologic and non-pharmacologic, physical, cognitive, and behavioral treatments, as well as means for disease prevention. - Independently perform comprehensive and specific physiatric examinations. - Perform diagnostic and treatment procedures common to the practice of PRM, such as electrodiagnostic medicine, MSK ultrasonography, and peripheral and axial injections, with either synchronous or asynchronous remote guidance by a consultant. - Prescribe different kinds of exercise.   ***Total for the Domain*** |  |  |  |  |  |
| **Interpersonal and Communication Skills**   - Demonstrate interpersonal and communication skills that result in effective exchange of information and collaboration with patients, their families, and other health professionals. - Exhibit effective and appropriate communication with patients, families, and the public across different socioeconomic and cultural backgrounds. - Work effectively as a member or leader of a healthcare team or other professional group, and act as a consultative role to other physicians or health professionals. - Maintain comprehensive, timely, and understandable medical records.   ***Total for the Domain*** |  |  |  |  |  |
| **Practice- and Systems-Based Learning and Improvement**   - Observe and gain fundamental understanding of the types of patients served, referral patterns and services available in the continuum of rehabilitation care provided in various settings. These may include critical, acute, and sub-acute care units, skilled nursing facilities, sheltered workshops, and other vocational facilities, schools for persons with multiple disabilities (including deafness and blindness), independent living facilities for individuals with physical impairments, day hospitals, home health care services, primary care setting, as well as community-based rehabilitation. Introduction to these options for care may be made by on-site visits to some of these facilities as well as didactic lectures. Residents should be encouraged to interact with health care consumer groups and organizations in supervised working environments. - Identify the inclusion criteria for a physical/ cognitive rehabilitation program for a patient and criteria for discharge. - Identify the main relevant patient groups for a disabled person. - Read pertinent articles from recent evidence-based medical literature on the assessment and treatment of conditions seen in Rehabilitation Medicine and apply this knowledge to the current care of a patient. - Use information technology to appraise and assimilate evidence from scientific studies to enhance patient care outcomes. - Engage in and write a research under supervision and disseminate research findings. - Present a report/ lecture to the consultants, residents, and other members of the rehabilitation team on an aspect of interest in the assessment and treatment of conditions seen in Rehabilitation Medicine. - Discuss with the consultants, residents, and other members of the rehabilitation team the limitations in the current knowledge base of a particular condition and possible directions for future clinical research to improve knowledge in this area. - Demonstrate skill in supervising residents, medical students, and members of the rehabilitation team in information gathering, decision-making and patient management.   ***Total for the Domain*** |  |  |  |  |  |
| **Reintegration of People with Disabilities into Society**   - Identify resources of education and training for a disabled person and/or participate in the orientation. - Identify resources of professional rehabilitation and/or participate in the orientation for reintegration. - Advocate for quality patient care and optimal patient care systems. - Identify the health, social and financial barriers, and possible resources. - Identify and establish the means allowing a disabled person to remain at home.   ***Total for the Domain*** |  |  |  |  |  |
| **Medical Ethics and Public Health**   - Identify individual and collective issues of public health and ethics related to disabled people. - Identify clinical situations (during rehabilitation) of unreasonable obstinacy related to care. - Conduct multi-professional discussions aiming at care limitations with the patient and relatives/ caregivers. - Incorporate considerations of cost awareness and risk-benefit analysis in patient and/or population-based care as appropriate.   ***Total for the Domain*** |  |  |  |  |  |
| **Quality Assurance**   - Participate in identifying system errors and implementing potential systems solutions. - Receive formal instruction regarding the principles, objectives and process of performance improvement and program evaluation, risk management, and cost-effectiveness in Medicine.   ***Total for the Domain*** |  |  |  |  |  |
| **Policies of Care and Prevention for Disabled People**   - Log PRM clinical activities and practical procedures. - Participate in public information about prevention and care for the main disabling diseases and the social integration of disabled people.   ***Total for the Domain*** |  |  |  |  |  |
| **Professionalism**   - Demonstrate a commitment to carry out professional responsibilities and an adherence to ethical principles. - Demonstrate compassion, integrity, and respect for others - Respect for patient privacy and autonomy. - Demonstrate responsiveness to patient needs that supersedes self-interest. - Demonstrate accountability to patients, society, and the profession. - Be sensitive and responsive to a diverse patient population, including, but not limited to diversity in gender, age, culture, race, religion, disabilities, and sexual orientation.   ***Total for the Domain*** |  |  |  |  |  |

*Based on the Core Curriculum and Competency recommended by the International Society of Physical and Rehabilitation Medicine and the Philippine Board of Rehabilitation Medicine.

[4] Strongly Agree; [3] Agree; [2] Disagree; [1] Strongly Disagree; [0] Not applicable.

Table 3. Residents’ self-evaluation of learning of PRM topics and skills through telerehabilitation (N = 30).

| **TOPIC** | ***Learning content as it relates to telerehabilitation experience*** | **D** | **P** | **I** | **NA** |
| --- | --- | --- | --- | --- | --- |
|  |  | n (%) | n (%) | n (%) | n (%) |
| **FUNDAMENTALS** | | | | | |
| **Rehabilitation Goals** | Broad definition of rehabilitation goals |  |  |  |  |
|  | Definition of short, medium and long term goals |  |  |  |  |
|  | History & Physical examination in PRM |  |  |  |  |
|  | ***Subtotal*** | $\Sigma$ (%) | $\Sigma$ (%) | $\Sigma$ (%) | $\Sigma$ (%) |
| **Rehabilitation theoretical background** | Sensory and Motor control systems |  |  |  |  |
|  | Posture and Balance control systems |  |  |  |  |
|  | Motor learning principles |  |  |  |  |
|  | Neuroplasticity |  |  |  |  |
|  | Mirror neuron paradigm |  |  |  |  |
|  | Principles of cardiovascular fitness |  |  |  |  |
|  | Principles of Kinesiology |  |  |  |  |
|  | Mechanisms of Respiration |  |  |  |  |
|  | Mechanisms of Excretion |  |  |  |  |
|  | Mechanisms of Swallowing |  |  |  |  |
|  | Physiology of aging |  |  |  |  |
|  | ***Subtotal*** | $\Sigma$ (%) | $\Sigma$ (%) | $\Sigma$ (%) | $\Sigma$ (%) |
| **WHO-ICF** | Definition of functioning and health |  |  |  |  |
|  | Classification in detail: Body Structure and Functions, Activity and Participation, Environmental factors, Capacity and Performance concepts |  |  |  |  |
|  | Check-list and Core-sets |  |  |  |  |
|  | ICF-Cy |  |  |  |  |
|  | ICF application to epidemiological research |  |  |  |  |
|  | ***Subtotal*** | $\Sigma$ (%) | $\Sigma$ (%) | $\Sigma$ (%) | $\Sigma$ (%) |
| **Functional assessment and outcome measurement** | Psychometric properties of clinical measures (accuracy, reliability, validity, feasibility, ceiling and floor effect, transcultural validation) |  |  |  |  |
|  | Classification of clinical outcome measures |  |  |  |  |
|  | (Category, Ordinal, Interval scales) |  |  |  |  |
|  | Pain measurements (VAS, NRS, Mc Gill Pain questionnaire,…) |  |  |  |  |
|  | Range of motion (goniometric measures) assessment |  |  |  |  |
|  | Muscle strength (Medical Research Council scale,…..) assessment |  |  |  |  |
|  | Motor impairment measures (examples, Motricity index, FuglMeyer,….) |  |  |  |  |
|  | Esteroception and proprioception assessment |  |  |  |  |
|  | Cranial nerve assessment |  |  |  |  |
|  | Bedside swallow assessment |  |  |  |  |
|  | ADL measures (examples , Barthel Index, Functional Assessment Measure) |  |  |  |  |
|  | Trunk control assessment (examples, Trunk control test, ….) |  |  |  |  |
|  | Gait measures (examples, Functional Ambulation Category, Hauser index, 10 m walking test) |  |  |  |  |
|  | Balance measures (examples, Tinetti scale, Berg Balance scale, Timed Up and Go) |  |  |  |  |
|  | Endurance measures (Borg scale, 6 m walking test,…) |  |  |  |  |
|  | Dexterity measures (examples: ARA test, Wolf MFT, Nine hole peg test, Motor Activity Log,.) |  |  |  |  |
|  | Cognition assessment: general (examples, Mini Mental state exam, Frontal Assessment battery, ) |  |  |  |  |
|  | Cognition assessment: focal (examples, Memory tests, Attention tests, Visuospatial ability tests, Frenchay Aphasia Screening test …) |  |  |  |  |
|  | Spasticity (examples Modified Ashworth scale, Tardieu scale, Goal Attainment score…..) |  |  |  |  |
|  | Speech |  |  |  |  |
|  | Consciousness Level |  |  |  |  |
|  | Sensory Disturbance |  |  |  |  |
|  | Quality of life (SF-36, Euroqol, …..) |  |  |  |  |
|  | Community reintegration inventories (i.e., Community Integration Questionnaire, Functional Status Questionnaire..) |  |  |  |  |
|  | Disease-specific scales (specific disability assessment for people with Neurological disorders, Musculoskeletal disorders, Cancer ,..) |  |  |  |  |
|  | ***Subtotal*** | $\Sigma$ (%) | $\Sigma$ (%) | $\Sigma$ (%) | $\Sigma$ (%) |
| **DIAGNOSTICS (knowledge includes: indications to the investigation, interpretation of major findings)** | Ultrasound assessment of joints and muscles |  |  |  |  |
|  | Ultrasound assessment of artero-venous vessels |  |  |  |  |
|  | Skeletal X-ray |  |  |  |  |
|  | Chest and abdominal X-ray |  |  |  |  |
|  | Brain CT |  |  |  |  |
|  | Brain MRI |  |  |  |  |
|  | Spine CT |  |  |  |  |
|  | Spine MRI |  |  |  |  |
|  | Chest and abdominal CT |  |  |  |  |
|  | Scintigram |  |  |  |  |
|  | Bone Mineral Density |  |  |  |  |
|  | EMG-ENG |  |  |  |  |
|  | EEG |  |  |  |  |
|  | Evoked potentials (MEPs, SSEPs, BAEPs,..) |  |  |  |  |
|  | Videofluoroscopy, Fiberoptic endoscopic evaluation of swallowing |  |  |  |  |
|  | Gait analysis (sEMG, kinetics) |  |  |  |  |
|  | Posturography |  |  |  |  |
|  | Motion analysis |  |  |  |  |
|  | ***Subtotal*** | $\Sigma$ (%) | $\Sigma$ (%) | $\Sigma$ (%) | $\Sigma$ (%) |
| **INTERVENTIONS (indications, efficacy, side effects)** | Education, psychological support to increase resilience/coping with disabilities |  |  |  |  |
|  | Drug treatment – analgesic, opioids, anticonvulsant |  |  |  |  |
|  | Drug treatments- anti-inflammation treatment |  |  |  |  |
|  | Drug treatment – oral and local spasticity treatment (botulinum toxin, phenol nerve blocks) |  |  |  |  |
|  | Drug treatment- antidepressant agents |  |  |  |  |
|  | Drug treatment- excretory disturbance |  |  |  |  |
|  | Therapeutic exercise, aquatherapy |  |  |  |  |
|  | Physical modalities, massage, manipulation, traction |  |  |  |  |
|  | Electrical stimulation (Transcutaneous, Functional /Neuromuscular) |  |  |  |  |
|  | Biofeedback |  |  |  |  |
|  | Neurodevelopmental approaches (Bobath, Kabat, Vojta, Rood,……) |  |  |  |  |
|  | Task-oriented approaches |  |  |  |  |
|  | Neurocognitive approach according to Perfetti |  |  |  |  |
|  | Action observation techniques |  |  |  |  |
|  | Mirror therapy |  |  |  |  |
|  | Non invasive cortical stimulation techniques |  |  |  |  |
|  | Orthotics and prosthetics |  |  |  |  |
|  | Locomotion aids/adaptive equipments (canes, rollators, power/manual wheelchairs, bath equipments) |  |  |  |  |
|  | Assistive technology , Augmentative communication device |  |  |  |  |
|  | Ergonomic Considerations in House, Workplace |  |  |  |  |
|  | Vocational Rehabilitation |  |  |  |  |
|  | Sport therapy |  |  |  |  |
|  | Music therapy, art therapy, pet therapy and other complementary/alternative medicine |  |  |  |  |
|  | Balneology, SPA therapy |  |  |  |  |
|  | Manual medicine |  |  |  |  |
|  | Acupuncture |  |  |  |  |
|  | ***Subtotal*** | $\Sigma$ (%) | $\Sigma$ (%) | $\Sigma$ (%) | $\Sigma$ (%) |
| **RESEARCH IN REHABILITATION** | Principles of epidemiology, quantitative and qualitative research |  |  |  |  |
|  | Research study design (experimental and observational studies, single-case studies, metanalysis and reviews) |  |  |  |  |
|  | Fundamentals of descriptive statistics (mean, SD, variance, confidence intervals, median, range, interquartile range; normal distribution) |  |  |  |  |
|  | Fundamentals of inferential statistics (independent and dependent variables, comparison statistical analysis for parametric and non parametric measures) |  |  |  |  |
|  | Sample size computation |  |  |  |  |
|  | Reporting results in graphics and tables, narrative assessment of outcome |  |  |  |  |
|  | Quality Improvement |  |  |  |  |
|  | ***Subtotal*** | $\Sigma$ (%) | $\Sigma$ (%) | $\Sigma$ (%) | $\Sigma$ (%) |
| **REHABILITATION APPROACHES TO DISEASE-SPECIFIC DISABILITIES**  **(Pathogenesis, clinical assessment, rehabilitation techniques, prognostic factors of recovery)** | | | | | |
| **Nervous system disorders** | Stroke |  |  |  |  |
|  | Acquired brain injury in adult age |  |  |  |  |
|  | Acquired brain injury in developmental age |  |  |  |  |
|  | Spinal cord injury (traumatic and not traumatic) in adults |  |  |  |  |
|  | Spinal cord injury (traumatic and not traumatic) in children |  |  |  |  |
|  | Autoimmune and inflammatory neurological conditions (e.g. Multiple Sclerosis) |  |  |  |  |
|  | Movement disorders (as a composite clinical condition) |  |  |  |  |
|  | Parkinson’s disease |  |  |  |  |
|  | Huntington disease |  |  |  |  |
|  | Dystonias |  |  |  |  |
|  | Peripheral Nervous System disorders and injuries (as a composite clinical condition) |  |  |  |  |
|  | Neuropathies |  |  |  |  |
|  | Myopathies/Dystrophies |  |  |  |  |
|  | Neuromuscular diseases in adults (including post-polio syndrome) |  |  |  |  |
|  | Neuromuscular diseases in developmental age (SMA, botulism) |  |  |  |  |
|  | Focal disorders of cognition and behaviour |  |  |  |  |
|  | Language and Speech disorders |  |  |  |  |
|  | Swallowing disorders |  |  |  |  |
|  | Cerebral palsy during the developmental age (including normal growth & development) |  |  |  |  |
|  | Cerebral palsy in the grown subjects |  |  |  |  |
|  | Congenital disorders of Nervous System (e.g. spina bifida, Arnold-Chiari malformation and so on) |  |  |  |  |
|  | Specific learning disorders in children (e.g. dyslexia, ADHD, mental retardation, dyspraxia….) |  |  |  |  |
|  | ***Subtotal*** | $\Sigma$ (%) | $\Sigma$ (%) | $\Sigma$ (%) | $\Sigma$ (%) |
| **Orthopedic and Musculoskeletal Disorders** | Osteoarthritis, crystal arthritis and degenerative musculoskeletal conditions |  |  |  |  |
|  | Hand Injuries |  |  |  |  |
|  | Musculoskeletal injuries |  |  |  |  |
|  | Limb loss (including congenital causes) and Amputations |  |  |  |  |
|  | Osteoporosis |  |  |  |  |
|  | Widespread pain syndromes (fibromyalgia, chronic fatigue syndrome, etc) and work-related musculoskeletal disorders/Industrial rehab |  |  |  |  |
|  | Spinal Disorders (including back pain, scoliosis) |  |  |  |  |
|  | Inflammatory and autoimmune disorders, joint & connective tissue disorders (e.g. Rheumatoid Arthritis and SLE, etc.) |  |  |  |  |
|  | Rehabilitation after joint prosthesis |  |  |  |  |
|  | Temporomandibular joint disorders |  |  |  |  |
|  | Sports Medicine |  |  |  |  |
|  | ***Subtotal*** | $\Sigma$ (%) | $\Sigma$ (%) | $\Sigma$ (%) | $\Sigma$ (%) |
| **Other Specific Disabling Conditions** | Pain management: Complex regional pain syndromes / Central pain / Neuropathic pain disorders/ Craniofacial pain/ Pelvic pain/ |  |  |  |  |
|  | Respiratory Diseases, ventilation failure (Pulmonary Rehabilitation) |  |  |  |  |
|  | Heart diseases (Cardiac Rehabilitation) |  |  |  |  |
|  | Peripheral Artery Diseases |  |  |  |  |
|  | Cancer : Oncological rehabilitation / |  |  |  |  |
|  | The frail patient (including the immobile patient); management of |  |  |  |  |
|  | Deconditioning, DVT prophylaxis/Treatment in Rehab patients |  |  |  |  |
|  | Postural Instability and Recurrent Falls |  |  |  |  |
|  | Wound Care (promotion of tissue viability, prevention and treatment of Pressure Sores) |  |  |  |  |
|  | Bladder and Bowel Disorders (perineal rehabilitation) |  |  |  |  |
|  | Sexual disorders (rehabilitation after spine lesion) |  |  |  |  |
|  | Disability after organ transplantation |  |  |  |  |
|  | Vascular Insufficiency/Dse, Lymphedema |  |  |  |  |
|  | Disability following major burns |  |  |  |  |
|  | Miscellanea (disability linked to severe obesity, metabolic disorders, psychiatric disorders, hearing & visual disorders, …) |  |  |  |  |
|  | ***Subtotal*** | $\Sigma$ (%) | $\Sigma$ (%) | $\Sigma$ (%) | $\Sigma$ (%) |
| **INTEGRATIVE AND CLINICAL REHABILITATION SCIENCES** | | | | | |
| **Integrative and clinical rehabilitation sciences** | Comprehensive PRM Intervention definition |  |  |  |  |
|  | Administration and management |  |  |  |  |
|  | Research on best care including guidelines, organization, coordination, and education |  |  |  |  |
|  | Standards and guidelines for the provision of best care (including Evidence Based Medicine) in PRM |  |  |  |  |
|  | PRM quality management |  |  |  |  |
|  | Scientific education and training of professionals in PRM |  |  |  |  |
|  | Development and evaluation of the PRM team and multidisciplinary care |  |  |  |  |
|  | Community-based rehabilitation issues |  |  |  |  |
|  | Networks and pathways in PRM |  |  |  |  |
|  | ***Subtotal*** | $\Sigma$ (%) | $\Sigma$ (%) | $\Sigma$ (%) | $\Sigma$ (%) |
| **Life after residency (for senior trainees)** | Different practice settings /marketing your practice |  |  |  |  |
|  | Medical liability |  |  |  |  |
|  | Interview process |  |  |  |  |
|  | Billing & Collections |  |  |  |  |
|  | Employment contracts |  |  |  |  |
|  | ***Subtotal*** | $\Sigma$ (%) | $\Sigma$ (%) | $\Sigma$ (%) | $\Sigma$ (%) |

*Based on the Core Curriculum and Competency recommended by the International Society of Physical and Rehabilitation Medicine and the Philippine Board of Rehabilitation Medicine.

[D] Demonstrated – able to perform skills without supervision; or able to apply theoretical knowledge in PRM practice; [P] Practiced – able to perform skills, but needs supervision; or needs guidance to apply theoretical knowledge in PRM practice; [I] Introduced – able to recognize the principles and processes of PRM skills; or able to discuss PRM theories and concepts; or [NA] Not applicable.

**Supplementary Material 3: Dummy Table for the Results of the Focus Group Discussion**

Gaps in the achievement of PRM competencies through telerehabilitation rated according to level of impact in each training institution (N=6).

| ***Gaps in PRM Competencies through Telerehabilitation according to Themes*** | ***PGH****  ***(n=3)*** | ***SLMC****  ***(n=3)*** | ***Total***  ***(N=6)*** |
| --- | --- | --- | --- |
| **Theme 1:**   - Gap 1 - Gap 2 | ***X***  ***X*** | ***X***  ***X*** | ***X***  ***X*** |
| **Theme 2:**   - Gap 1 - Gap 2 | ***X***  ***X*** | ***X***  ***X*** | ***X***  ***X*** |
| **Theme 3:**   - Gap 1 - Gap 2 | ***X***  ***X*** | ***X***  ***X*** | ***X***  ***X*** |

*Impact of the felt gap in the training institution on a scale of 0 (not felt at all) to 10 (very much felt). X: Mean. PGH: Philippine General Hospital. SLMC: St. Luke’s Medical Center - Quezon City.
